# Influence of Dislocations on the Refractive Index of AlN by Nanoscale Strain Field

**DOI:** 10.1186/s11671-019-3018-7

**Published:** 2019-05-30

**Authors:** Jianwei Ben, Xiaojuan Sun, Yuping Jia, Ke Jiang, Zhiming Shi, You Wu, Cuihong Kai, Yong Wang, Xuguang Luo, Zhe Chuan Feng, Dabing Li

**Affiliations:** 1 State Key Laboratory of Luminescence and Applications, Changchun Institute of Optics, Fine Mechanics and Physics, Chinese Academy of Sciences, Changchun, 130033 China; 2 Center of Materials Science and Optoelectronics Engineering, University of Chinese Academy of Sciences, Beijing, 100049 China; 3 Laboratory of Optoelectronic Materials and Detection Technology, Guangxi Key Laboratory for the Relativistic Astrophysics, School of Physical Science and Technology, Guangxi University, Nanning, 530004 China

**Keywords:** Refractive index, AlN, Threading dislocation density, Nanoscale strain field around dislocations

## Abstract

The refractive index of AlN has a direct influence on AlGaN-based deep ultraviolet optoelectronic devices, such as the external quantum efficiency of light-emitting devices. Revealing the dependence of the refractive index of AlN on the threading dislocations is meaningful since high-density threading dislocations usually exist in AlN. In this paper, the effect of different dislocation densities on the refractive index of AlN is investigated. With the increase of dislocation densities from 4.24 × 10^8^ to 3.48 × 10^9^ cm^− 2^, the refractive index of AlN decreases from 2.2508 to 2.2102 at 280 nm. Further study demonstrates that the nanoscale strain field around dislocations changes the propagation of light and thus decreases the refractive index of AlN. This study will be beneficial to the design of optoelectronic devices and thus realizing high-performance deep ultraviolet optoelectronic devices.

**Electronic supplementary material:**

The online version of this article (10.1186/s11671-019-3018-7) contains supplementary material, which is available to authorized users.

## Introduction

AlN-based materials are promising materials to fabricate deep ultraviolet (DUV) optoelectronic devices such as light-emitting diodes (LEDs) [1–5], laser diodes [6–8], and photodetectors [9, 10] due to the direct bandgap tunable from 3.4 to 6.2 eV [11]. The refractive index of AlN has effects on the performance of the optoelectronic devices directly. For LEDs, the refractive index of AlN has impacts on the light extract efficiency (LEE) because the total internal reflection angle is determined by the difference of the refractive index between the AlN layer and other region, which is the key limiting factor for the amount of light output. Since the external quantum efficiency (EQE) is the product of the internal quantum efficiency and the LEE, the refractive index of AlN will affect the EQE of LEDs. Also, the refractive index plays a key role in the design of waveguide structures such as distributed Bragg reflector (DBR) [12–14], the reflectivity of which is sensitive to the refractive index. Therefore, revealing the factors that affect the refractive index of AlN is important.It can be learned from previous studies that the refractive index of AlN can be affected by many factors including temperature, pressure, and bandgap. The refractive index of AlN increases with higher temperature [15] and lower pressure [16]. For AlN-based material, the refractive index becomes lower with the increase of bandgap [17]. Also, the dislocations in semiconductors have great influence on the properties of semiconductors and the performance of devices. The dislocations will release the stress in materials [18]. They also will affect the dark current and responsivity of the photodetectors [19] and influence the IQE of multiple quantum wells [11, 20] and so on. However, few researches focus on the influence of different threading dislocation densities (TDDs) on the refractive index of AlN, though there are high TDDs in AlN materials, which usually vary from 10^8^ to 10^9^ cm^− 2^ orders from recent reports [21–23]. Investigating on the correlation between TDDs and the refractive index of AlN is the key to optimize the performance of the optoelectronic devices. In this paper, the dependence of different TDDs on the refractive index of AlN has been studied. The different photon wavelengths are used, such as 633 nm, 365 nm, and 280 nm. The results show that the dislocations lead to the decrease of the refractive index of AlN. The results will benefit the design and simulation of AlN-based optoelectronic devices such as DUV LEDs and DBR structures.

## Methods

To study the relationship between dislocations and the refractive index of AlN, AlN templates were grown by metal-organic chemical vapor deposition (MOCVD) on c-sapphire substrates and then annealed at different temperatures to obtain AlN samples with different dislocation densities.

When growing AlN templates by MOCVD, trimethylaluminum and ammonia were used as precursor gases. Hydrogen was used as carrier gas. The pressure during growth was kept at 40 mbar. The growth temperature and time of nucleation layer is about 955 °C for 150 s and then raised to 1280 °C for high-temperature (HT) AlN growth. After 15-min high-temperature AlN growth, an AlN interlayer was grown at 1050 °C for 160 s. Finally, the growth temperature was raised to 1280 °C to grow thick HT AlN for 50 min. The total thickness of AlN film is about 1.1 μm.

After the growth of the AlN layer by MOCVD, the AlN templates were ex situ annealed at 1500 °C, 1600 °C, 1700 °C, and 1750 °C for 1 h, respectively. The AlN layer without annealing was marked as sample 1, and the samples after 1500 °C to 1750 °C annealing were marked as samples 2 to 5. The X-ray diffraction (XRD) was used to measure the TDDs in AlN samples, and the spectroscopic ellipsometric (SE) measurement was taken to measure the refractive index. The Raman shift spectra were adopted to characterize the stress state of AlN templates.

## Results and Discussion

**Fig. 1:**
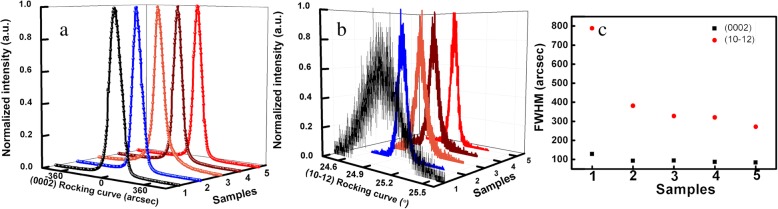
**a** The (0002) plane XRC of five AlN samples. **b** The (10-12) plane XRC of five AlN samples. **c** The FWHM of (0002, 10-12) plane XRC; the red circle means the FWHM of (10-12) plane and black square represents the FWHM of (0002) plane

Figure 1 a and b show the (0002) and (10-12) plane XRD rocking curves (XRC) of the five AlN samples. It can be observed that the full width at half maximum (FWHM) of (0002) plane XRC slightly decreases and the FWHM of (10–12) plane XRC greatly decreases from sample 1 to sample 5. The density of dislocations with screw and edge component can be calculated using the FWHM of (0002) and (10–12) plane XRC according to formula (1) and (2): [24, 25].

1 $$ {\rho}_{\mathrm{s}}={\beta_{(0002)}}^2/\left(2\pi \ln 2\times {\left|{b}_c\right|}^2\right) $$

2 $$ {\rho}_{\mathrm{e}}={\beta_{\left(10-12\right)}}^2/\left(2\pi \ln 2\times {\left|{b}_a\right|}^2\right) $$

where *ρ*_*s*_ and *ρ*_*e*_ represent the density of the dislocation with screw and edge component, respectively. *β* is the FWHM of XRC. |*b*_*c*_| equates to c-axial lattice constant, and |*b*_*a*_| equates to a-axial lattice constant of AlN. The FWHM of (0002) and (10–12) plane XRC are exhibited in Fig. 1c for the five AlN samples and the calculated TDDs of the five AlN samples are shown in Table 1.

**Table 1:** The TDDs, refractive index, and Raman *E*_2_(*h*) peak positions of AlN samples

Samples	1	2	3	4	5
Total TDDs (cm^− 2^)	3.48 × 10^9^	8.27 × 10^8^	6.15 × 10^8^	5.86 × 10^8^	4.24 × 10^8^
Refractive index at 633 nm	2.0190	2.0249	2.0312	2.0436	2.0559
Refractive index at 365 nm	2.1001	2.1076	2.1131	2.1264	2.1405
Refractive index at 280 nm	2.2102	2.2175	2.2222	2.2366	2.2508
Position of *E*_2_(*h*) (cm^−1^)	657.56	661.12	662.88	663.20	663.49

**Fig. 2:**
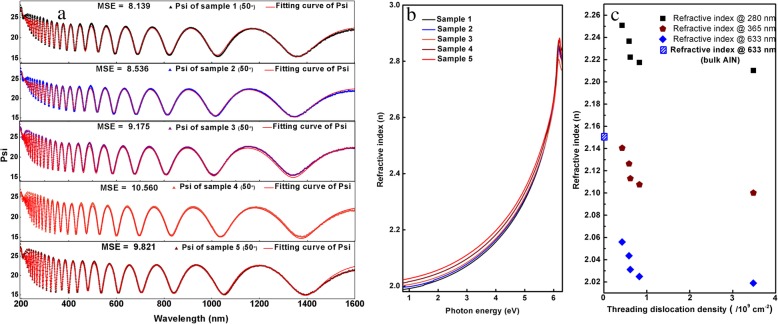
**a** Partial experimental data of SE measurement and fitting curves. **b** The refractive index curve. **c** Refractive index vs. different TDDs at 280 nm, 365 nm, and 633 nm

The SE experimental data of the five samples are fitted by CompleteEASE software (J.A. Woollam Inc.) using a parametric semiconductor model, which can reproduce the optical properties of direct band gap semiconductors effectively [26]. Figure 2 a shows partial experimental and fitting curves of the five samples. The mean-squared error (MSE) of the five samples is 8.139, 8.536, 9.175, 10.560, and 9.821, respectively, which confirms the good fitting results. All the data and fitting results are provided in Additional file 1.

The refractive index curves of the five samples can be obtained from fitting results as shown in Fig. 2b. When the photon energy is lower than the bandgap of AlN (about 6.2 eV), the refractive index increases with increasing the photon energy for all the five samples. However, when the photon energy is higher than 6.2 eV, the refractive index decreases with the increase of photon energy. This phenomenon can be described by Kramers–Krőnig dispersion relation. With the decrease of TDDs in AlN, the refractive index increases from 2.019 to 2.056 at 633 nm, which is closer to that of bulk AlN (2.15 at 633 nm [27]). It means that the dislocations in AlN make the refractive index smaller than that of bulk AlN crystal.

The relationship between the refractive index and TDDs at 4.42 eV (280 nm, solar blind UV), 3.40 eV (365 nm, bandgap of GaN), and 1.96 eV (633 nm) are shown in Fig. 2c as well as in Table 1. It can be seen that the refractive index of AlN decreases with the increase of TDDs. With the increase of dislocation densities from 4.24 × 10^8^ to 3.48 × 10^9^ cm^− 2^, the refractive index of AlN decreases from 2.2508 to 2.2102 at 280 nm.

To reveal the mechanism on how dislocations change the refractive index of AlN, the strain field induced by dislocations is studied. The relationship between the refractive index and the strain filed is described by formula (3) [28]:

3 $$ \Delta {\left(\frac{1}{n^2}\right)}_i= PS=\sum \limits_{ij}{p}_{ij}{s}_j $$

In the formula, *p*_ij_ are the elasto-optic tensor and *S* is the presence of strain. The photoelastic constants matrix *P* of wurtzite AlN is shown as expression (4) [29, 30].

4 $$ p=\left(\begin{array}{l}-0.1\kern1.75em -0.027\kern0.75em -0.019\kern1em 0\kern3em 0\kern2.75em 0\\ {}-0.027\kern0.5em -0.1\kern2em -0.019\kern1em 0\kern3em 0\kern2.75em 0\\ {}-0.019\kern0.5em -0.019\kern1em -0.107\kern1em 0\kern3em 0\kern2.75em 0\\ {}0\kern2.75em 0\kern3em 0\kern3.5em -0.032\kern0.75em 0\kern2.75em 0\\ {}0\kern2.75em 0\kern3em 0\kern3.5em 0\kern3em -0.032\kern0.5em 0\\ {}0\kern2.75em 0\kern3em 0\kern3.5em 0\kern3em 0\kern2.75em -0.037\end{array}\right) $$

**Fig. 3:**
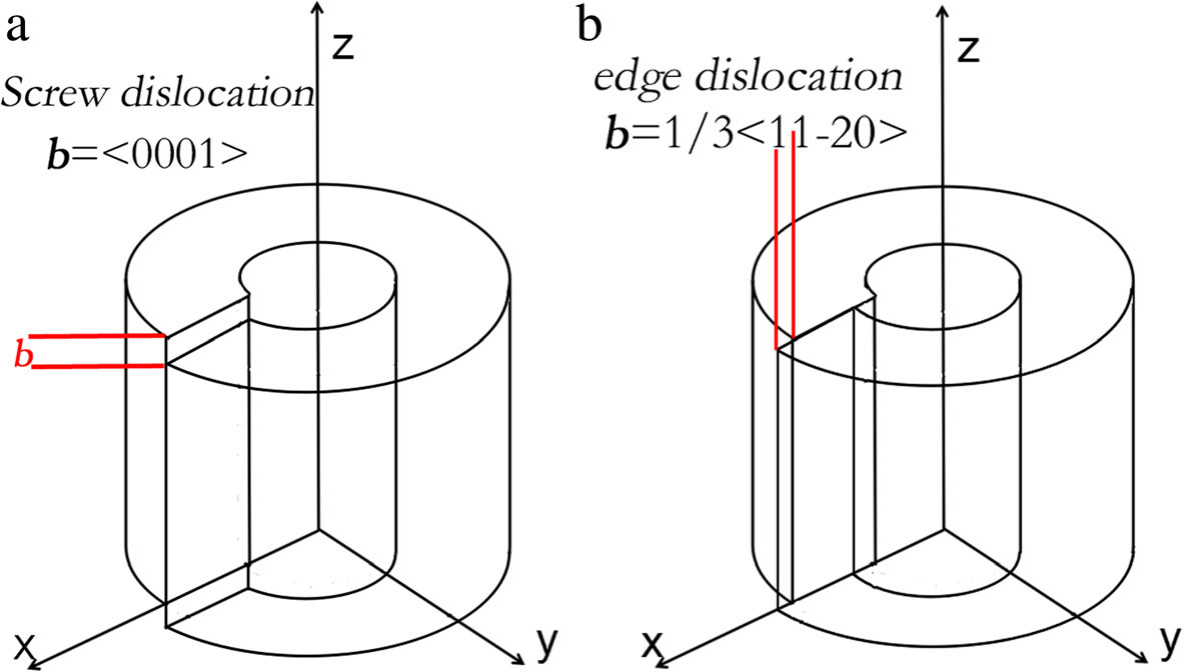
Cylindrical ring model of **a** screw dislocation and **b** edge dislocation

The strain field matrices of screw dislocation and edge dislocation in AlN are considered. The cylindrical ring models of the two kinds of dislocation are described in Fig. 3. According to the models, the distribution of the strain field around single dislocation can be obtained [31, 32].

The strain field around unit screw dislocation can be written as:

5 $$ {e}_{xz}={e}_{zx}=-\frac{b}{4\pi}\frac{y}{\left({x}^2+{y}^2\right)} $$

5a $$ {e}_{yz}={e}_{zy}=\frac{b}{4\pi}\frac{x}{\left({x}^2+{y}^2\right)} $$

5b $$ {e}_{xx}={e}_{yy}={e}_{zz}={e}_{xy}={e}_{yx}=0 $$

The strain field around unit edge dislocation can be written as:

6 $$ {e}_{xx}=-\frac{b}{4\pi \left(1-v\right)}\frac{y\left({x}^2-{y}^2\right)}{{\left({x}^2+{y}^2\right)}^2}-\frac{b}{2\pi}\frac{y}{\left({x}^2+{y}^2\right)} $$

6a $$ {e}_{yy}=\frac{b}{4\pi \left(1-v\right)}\frac{y\left(3{x}^2+{y}^2\right)}{{\left({x}^2+{y}^2\right)}^2}-\frac{b}{2\pi}\frac{y}{\left({x}^2+{y}^2\right)} $$

6b $$ {e}_{zz}=\frac{b\left(\lambda -2 v\lambda -2 Gv\right)}{2\pi \left(2G+\lambda \right)\left(1-v\right)}\frac{y}{x^2+{y}^2} $$

6c $$ {e}_{xy}={e}_{yx}=\frac{b}{4\pi \left(1-v\right)}\frac{x\left({x}^2-{y}^2\right)}{{\left({x}^2+{y}^2\right)}^2} $$

6d $$ {e}_{xz}={e}_{zx}={e}_{yz}={e}_{zy}=0 $$

where *b* is the length of the Burgers vector of unit dislocation and *e* represents the strain around the dislocation. *G* = 121 GPa is the shear modulus of wurtzite AlN; *λ* = 117.1 GPa and *v* = 0.241 are the lame constant and Poisson’s ratio [33, 34], respectively. According to the correspondence between *e*_*ij*_ and *S*_*k*_ (*i*,*j* = *x*,*y*,*z*; *k* = 1,2,3...6) [35], we convert the strain field into matrix formation as below to further present the change of refractive index caused by the dislocations.

7 $$ {S}_{\mathrm{edge}}=\left({S}_1\kern0.5em {S}_2\kern0.5em {S}_3\kern0.5em 0\kern0.5em 0\kern0.5em {S}_6\right) $$

8 $$ {S}_{\mathrm{screw}}=\left(0\kern0.5em 0\kern0.5em 0\kern0.5em {S}_4\ {S}_5\kern0.5em 0\right) $$

Taken the matrices (7) and (8) into formula (3), we can get the expression of Δ*n* caused by unit screw and unit edge dislocation.

9 $$ \Delta {\left(\frac{1}{n^2}\right)}_{\mathrm{screw}}={\left(\frac{1}{n_1^2}-\frac{1}{n_0^2}\right)}_{\mathrm{screw}}=-0.032\left({S}_4+{S}_5\right)=-0.008\frac{b\left(x-y\right)}{\pi \left({x}^2+{y}^2\right)} $$

10 $$ \Delta {\left(\frac{1}{n^2}\right)}_{\mathrm{edge}}={\left(\frac{1}{n_1^2}-\frac{1}{n_0^2}\right)}_{\mathrm{edge}}=-0.146\left({S}_1+{S}_2\right)-0.145{S}_3-0.037{S}_6=\hbox{-} 0.146\left(\frac{b}{4\pi \left(1-v\right)}-\frac{b}{2\pi}\right)\frac{2y}{x^2+{y}^2}-0.145\frac{b\left(\lambda -2\lambda v-2 Gv\right)}{2\pi \left(2G+\lambda \right)\left(1-v\right)}\frac{y}{x^2+{y}^2}-0.037\frac{b}{4\pi \left(1-v\right)}\frac{x\left({x}^2-{y}^2\right)}{{\left({x}^2+{y}^2\right)}^2} $$

**Fig. 4:**
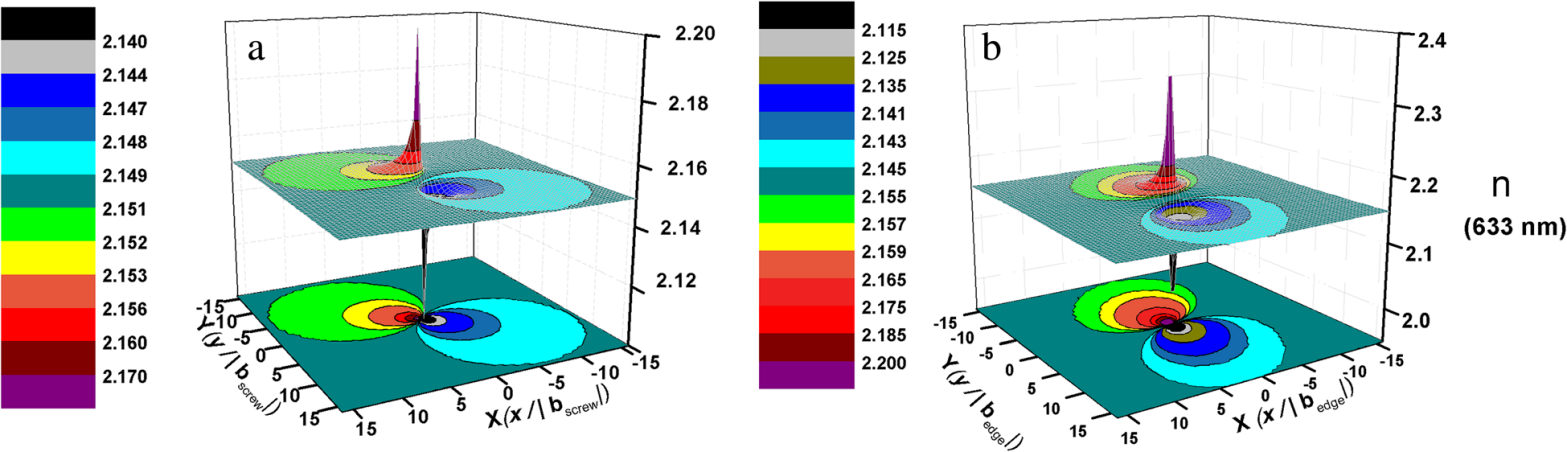
The distribution of refractive index at 633 nm around **a** unit screw dislocation and **b** unit edge dislocation

Based on the calculation, the distributions of refractive index (take the refractive index at 633 nm as an example) around unit screw and unit edge dislocations are shown in Fig. 4. It exhibits that the refractive index around the dislocation changes along the radial direction from the dislocation core which can be regarded as an inhomogeneous medium. Thus, light propagating in AlN will be correspondently influenced by TDDs. Scattering and interference will happen [36] when light goes through these refractive fields around dislocations. As a result, the refractive index of AlN will be changed, which is correspondence with the scattering matrix of the inhomogeneous medium [37].

**Fig. 5:**
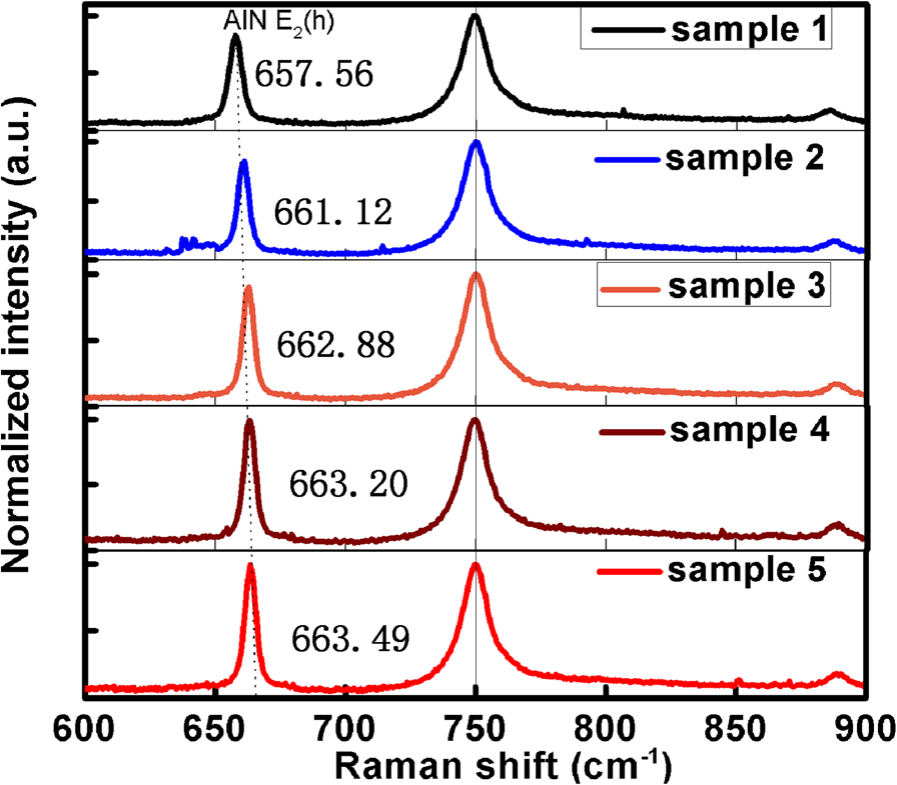
Raman shift spectra of the five samples

As mentioned in the “Introduction” section, other influence factors should be obviated to prove that the refractive index is truly influenced by dislocations. All the samples were measured at room temperature to obviate the influence of temperature. To obviate the influence of the stress in AlN material, Raman spectrum was taken to confirm the stress in AlN and the results are shown in Fig. 5. The *E*_*g*_ mode peak of sapphire at 750 cm^− 1^ is taken as calibration. The Raman shift peak of AlN *E*_2_(*h*) blue shifts with the decrease of TDDs as shown in Table 1. The blue shift of *E*_2_(*h*) peak means the AlN suffers more and more compressive stress from sapphire substrate. However, with the increase of compressive stress, the refractive index becomes closer to that of bulk AlN at 633 nm. It can be clearly obtained that the stress of AlN suffers from heterogeneous substrates has little influence on the refractive index. Additional evidence to support the conclusion is that the refractive index of AlN is also smaller than that of bulk AlN when AlN suffers tensile stress from the Si substrate [38], which is the same to the condition that AlN suffers compressive stress in this work. This phenomenon can be attributed to the fact that the stress of AlN suffers from substrates is too small to make a significant change on the refractive index of AlN. As a result, compared to the influence of other factors, the effect of the stress from substrates on the refractive index of AlN can be neglected.

The bandgap of the five samples is also calculated here. The optical absorption coefficient *α* is extracted from SE fitting results, and then the bandgap *E*_*g*_ is calculated based on the formula below [39]:

11 $$ {\left(\alpha E\right)}^2=\left\{\begin{array}{c}C\left(E-{E}_g\right)\kern0.75em \left(E\ge {E}_g\right)\\ {}0\kern4.75em \left(E<{E}_g\right)\end{array}\right. $$

**Fig. 6:**
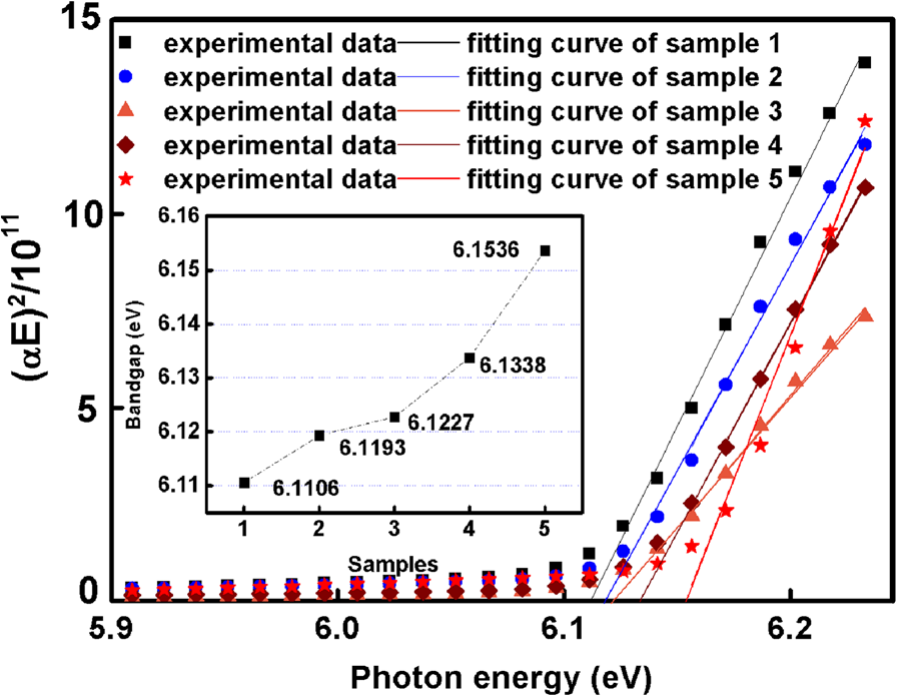
Dependence of (*αE*)^2^ on (*E*), the inset picture shows the bandgap of AlN templates

The plot of (*αE*)^2^ vs. *E* is presented as Fig. 6. The intercept of the *x*-axis is the value of *E*_*g*_. From the intercept of fitting curves on the *x*-axis, the increasing bandgap from 6.1106 to 6.1536 eV for sample 1 to sample 5 is shown in Fig. 6. The relationship between the refractive index and the bandgap is shown as below [16].

12 $$ n(E)={\left[a{\left(\frac{E}{E_g}\right)}^2\left(2-{\left(1+\frac{E}{E_g}\right)}^{0.5}-{\left(1-\frac{E}{E_g}\right)}^{0.5}\right)+b\right]}^{0.5} $$

where *E* is the photon energy and *E*_*g*_ is the bandgap of AlN. *a* and *b* are constants which equal to 13.70 and 7.81 for AlN, respectively. The refractive index of AlN should decrease with the increase of *E*_*g*_ according to the formula. However, in this work, the refractive index of AlN increases with the increase of *E*_*g*_, which means the influence of the bandgap on the refractive index of AlN can be neglected compared to the influence of TDDs. Therefore, the change of TDDs plays a key role in the change of the refractive index of AlN.

Combined with the above analyses, it is confirmed that the nanoscale strain field will influence the distribution of refractive index around dislocations, which further influence the refractive index of AlN. The dislocations will decrease the refractive of AlN according to the experimental data.

## Conclusions

In conclusion, the effect of TDDs on the refractive index of AlN is studied both experimentally and theoretically. Obviating the influence of temperature, stress, and bandgap, the conclusion can be obtained that the refractive index of AlN decreases with the increase of TDDs. Further studies showed that the nanoscale strain field around dislocations results in the refractive index changing significantly around the dislocations. Scattering and interference will occur once light propagates through dislocations and thus the refractive index of AlN will be changed. The findings in this work will be beneficial to optimizing AlN-based DUV optoelectronic devices.

## Additional file

Additional file 1: **Figure S1**. The fitting of sample 1, the mean-squared error is 8.139. **Figure S2**. The fitting of sample 2, the mean-squared error is 8.536. **Figure S3**. The fitting of sample 3, the mean-squared error is 9.175. **Figure S4**. The fitting of sample 4, the mean-squared error is 10.560. Figure S5. The fitting of sample 5, the mean-squared error is 9.821. (DOC 2035 kb)

## Data Availability

All data can be provided on a suitable request.

## Abbreviations

DBR: Distributed Bragg reflector
DUV: Deep ultraviolet
EQE: External quantum efficiency
FWHM: Full width at half maximum
LEDs: Light-emitting diodes
LEE: Light extract efficiency
MOCVD: Metal-organic chemical vapor deposition
MSE: Mean-squared error
SE: Spectroscopic ellipsometric
TDDs: Threading dislocation densities
XRC: XRD rocking curve
XRD: X-ray diffraction
