# Supplementary material for: Influence of Dislocations on the Refractive Index of AlN by Nanoscale Strain Field
Source: Nanoscale Res Lett. 2019 May 30;14:184. doi: 10.1186/s11671-019-3018-7 (PMC6542867; doi:10.1186/s11671-019-3018-7)
Supplement: Supplementary file 1 — Figure S1. The fitting of sample 1, the mean-squared error is 8.139. Figure S2. The fitting of sample 2, the mean-squared error is 8.536. Figure S3. The fitting of sample 3, the mean-squared error is 9.175. Figure S4. The fitting of sample 4, the mean-squared error is 10.560. Figure S5. The fitting of sample 5, the mean-squared error is 9.821. (DOC 2035 kb) [file 11671_2019_3018_MOESM1_ESM.doc]

**Supplementary information:**

**Influence of dislocations on the refractive index of AlN by nanoscale strain field**

Jianwei Ben^1,2^, Xiaojuan Sun1,[[1]](#footnote-2)a), Yuping Jia^1^, Ke Jiang^1,2^, Zhiming Shi^1^, You Wu^1,2^, Cuihong Kai^1,2^, Yong Wang^1,2^, Xuguang Luo^3^, Zhe Chuan Feng^3^, and Dabing Li1,[[2]](#footnote-3)a)

^1^*State Key Laboratory of Luminescence and Applications, Changchun Institute of Optics, Fine Mechanics and Physics, Chinese Academy of Sciences, Changchun 130033, China*

^2^ *Center of Materials Science and Optoelectronics Engineering, University of Chinese Academy of Sciences, Beijing 100049 , China*

^3^ *Laboratory of Optoelectronic Materials & Detection Technology, Guangxi Key Laboratory for the Relativistic Astrophysics, School of Physical Science & Technology, Guangxi University, Nanning 530004, China*

The curves of fitting and original data are shown in this supplementary information. The red and green lines are the Psi and Delta experimental curves for 50 to 70 degree, respectively. The dotted lines are the fitting curves.


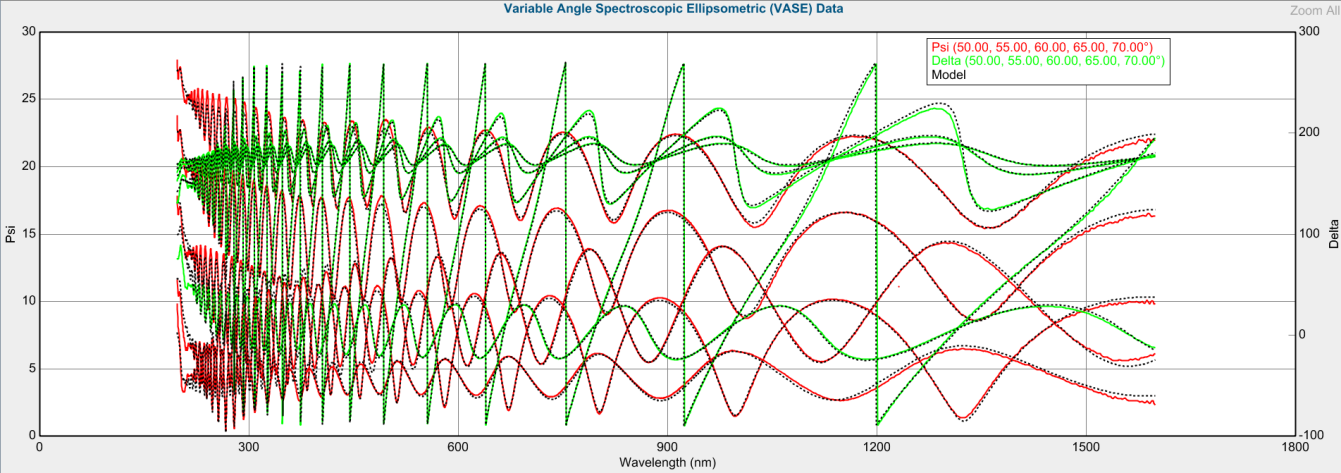


Figure S1. The fitting of sample 1, the mean-squared error is 8.139.


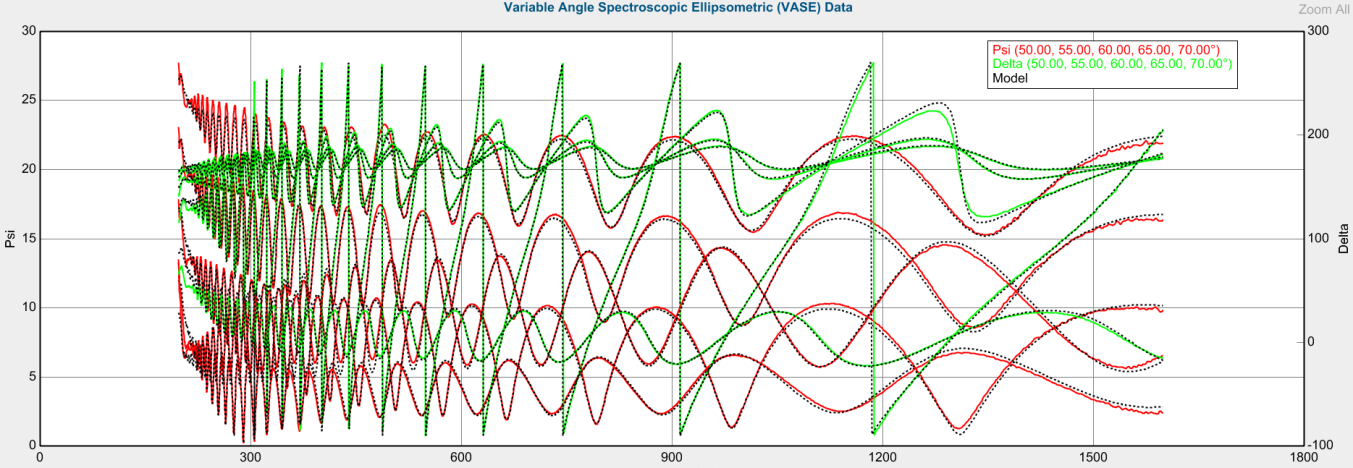


Figure. S2. The fitting of sample 2, the mean-squared error is 8.536.


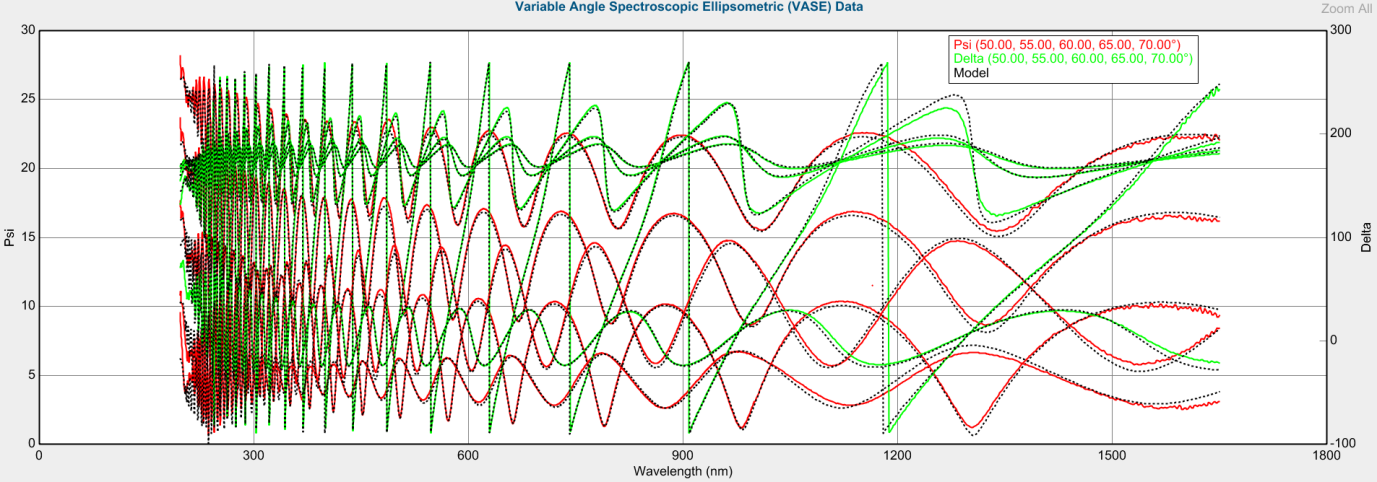


Figure. S3. The fitting of sample 3, the mean-squared error is 9.175.


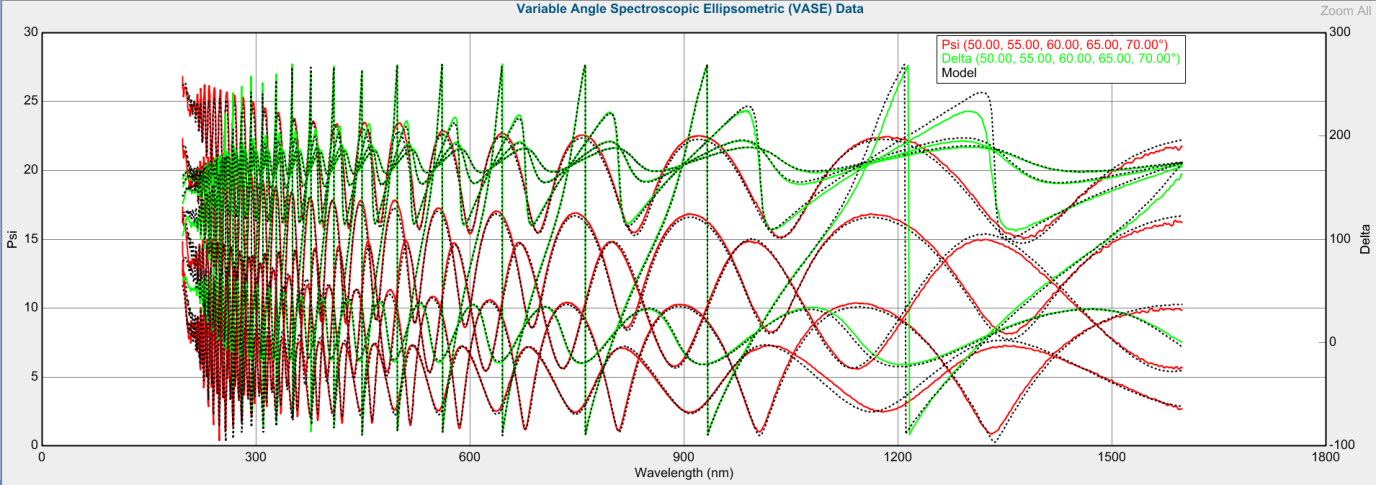


Figure. S4. The fitting of sample 4, the mean-squared error is 10.560.


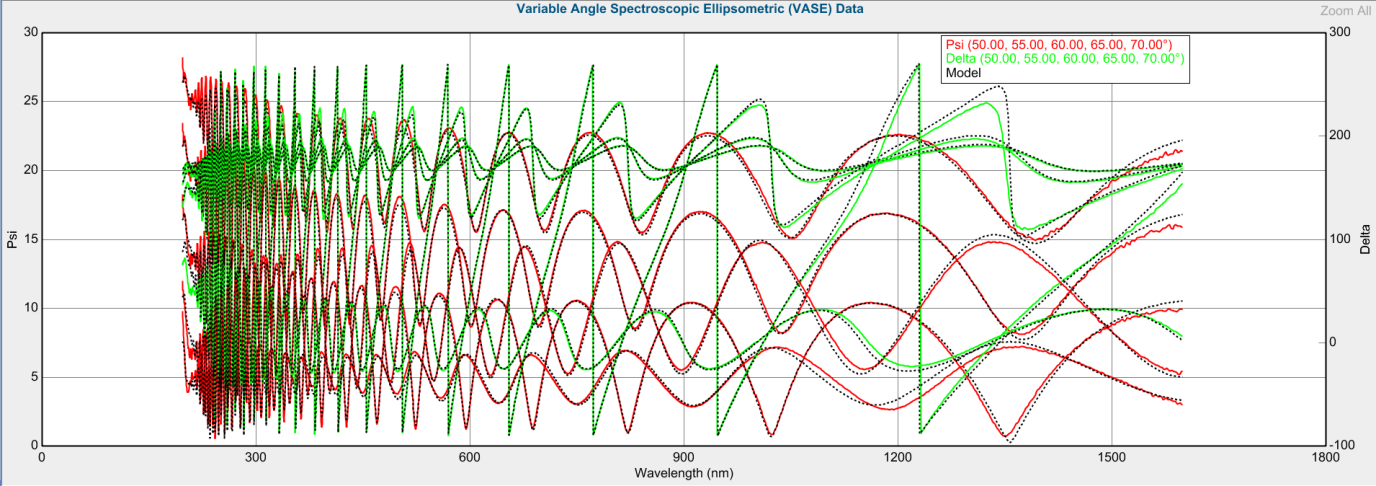


Figure. S5. The fitting of sample 5, the mean-squared error is 9.821.

1. ^a)^ Corresponding E-mail: [sunxj@ciomp.ac.cn;](mailto:sunxj@ciomp.ac.cn;) [lidb@ciomp.ac.cn](mailto:lidb@ciomp.ac.cn). [↑](#footnote-ref-2)
2. ^a^ [↑](#footnote-ref-3)
